# Midwives’ Attitudes Toward and Experience With a Tablet Intervention to Promote Safety Behaviors for Pregnant Women Reporting Intimate Partner Violence: Qualitative Study

**DOI:** 10.2196/16828

**Published:** 2020-05-20

**Authors:** Lisa Garnweidner-Holme, Lena Henriksen, Eva Marie Flaathen, Tone Klette Bøhler, Mirjam Lukasse

**Affiliations:** 1 Department of Nursing and Health Promotion Faculty of Health Sciences Oslo Metropolitan University Oslo Norway; 2 Faculty of Health and Social Sciences University of South-Eastern Norway, Campus Vestfold Borre Norway

**Keywords:** intimate partner violence, mHealth, attitudes, midwives, prenatal care

## Abstract

**Background:**

Violence against women is considered a global health problem, and intimate partner violence (IPV) around the time of childbirth can have severe consequences for mother and child. Prenatal care is considered a window of opportunity to address IPV and ask women about exposure to violence since women are in regular contact with health care providers. Mobile health (mHealth) interventions might overcome the barriers to talking about IPV face-to-face.

**Objective:**

Our objective was to explore midwives’ attitudes toward a tablet intervention consisting of information about IPV and safety behaviors as well as their experiences with recruiting pregnant women of different ethnic backgrounds in a randomized controlled trial (RCT).

**Methods:**

Individual interviews were conducted with 9 midwives who recruited participants for an RCT to test a video to promote safety behaviors delivered on a tablet during prenatal care. Analysis was guided by thematic analysis.

**Results:**

Midwives perceived the tablet intervention as an appropriate supplement during prenatal care to provide information about IPV and promote safety behaviors. They participated in the RCT primarily to obtain more knowledge regarding how to communicate about IPV. The intervention was perceived as an anonymous door-opener to talk about IPV and a good solution to ensure that every woman gets the same information. However, the content of the intervention had to be trustworthy and align with the information the midwives provide to women. Given the sensitivity of IPV, midwives outlined the importance of following the intervention with face-to-face communication. Midwives reported technical problems and a high demand on their time as the main challenges to recruiting women. They experienced challenges recruiting women of different ethnic backgrounds due to linguistic barriers and the women’s skepticism about scientific research.

**Conclusions:**

The tablet intervention might help midwives communicate about IPV. Although the video was considered as an anonymous door-opener to talk about IPV, midwives outlined the importance of following the intervention with face-to-face communication. The scarcity of midwives’ time during consultations has to be considered when implementing the intervention. Further research is needed to overcome barriers that limit inclusion of women from different ethnic backgrounds.

**Trial Registration:**

ClinicalTrials.gov NCT03397277; https://clinicaltrials.gov/ct2/show/NCT03397277

## Introduction

Intimate partner violence (IPV) is recognized as a global health problem [1]. According to the definition of the World Health Organization, IPV may include physical aggression, sexual coercion, psychological abuse, or controlling behaviors by current or former partners [1]. Approximately 30% of women worldwide have experienced IPV sometime during their lives [1]. Pregnancy does not protect women from violence. A meta-analysis of IPV during pregnancy that consisted of 92 studies from 23 countries reported an average prevalence of emotional abuse of 28.4%. The prevalences of physical abuse and sexual abuse were 13.8 and 8.0%, respectively [2]. In Norway, studies show the prevalence varies from 1% to 5% [3-5]. Although IPV occurs in all social strata, women with low education levels and women with limited economic resources are at higher risk [6]. Immigrant women are likely to be overrepresented in these groups; hence, they are more prone to be exposed to IPV [6]. The potential impact of IPV on a woman’s health prior to pregnancy, during pregnancy, and during the newborn period is well documented and includes depression, miscarriage, perinatal death, preterm birth, and low birth weight [7-9].

Interventions to reduce IPV during pregnancy are urgently needed. Prenatal care is recognized as an ideal window of opportunity to address IPV because this is a time when women are in regular contact with heath care providers [10]. As in many other countries, the Norwegian guidelines for prenatal care strongly recommend that health professionals routinely ask all pregnant women about their experiences with violence [11]. There is some evidence that screening for IPV in prenatal care might increase the identification of violence [12]. However, the evidence regarding how to assess and intervene amid violence during pregnancy and the postpartum period is inconclusive [13-16]. Qualitative studies can be useful in the evaluation of interventions to prevent IPV [17].

Studies among health professionals and women experiencing IPV report several barriers to communicating about IPV face-to-face in clinical settings [18-21]. Mobile health (mHealth) technology such as mobile phones, tablets, and other wireless computing devices may have the potential to overcome some of the barriers regarding face-to-face interventions [22,23]. Health professionals’ acceptance of mHealth interventions and willingness to recruit are crucial factors for successful mHealth interventions. Two systematic reviews on health professionals’ acceptance found divergent results [24,25]. White et al [24] concluded that health care professionals’ acceptance of using mHealth was generally very high. However, health care professionals’ acceptance of mHealth interventions during inpatient treatment was low [25]. The authors identified social norms, performance expectancy, and internet integration in the treatment course as the significant predictors for health professionals’ acceptance [25]. There is some evidence for the efficacy of Web-based safety decision aids for women experiencing IPV [26]; however, we have not found any published studies about health professionals’ acceptance of and experiences with mHealth interventions to prevent IPV during prenatal care.

This study was part of the Safe Pregnancy study, where a tablet intervention to prevent IPV was tested during a randomized controlled trial (RCT) during routine prenatal care at 19 maternal and child health centers (MCHCs) in southeastern Norway. The intervention in the Safe Pregnancy consisted of questions about IPV and a video with information about IPV and safety behaviors [27]. The intervention video lasted for 7 minutes. Only those women who had screened positive for IPV and were randomized to the intervention group saw the video about violence. Women in the control group saw a 7-minute video about a healthy and safe pregnancy in general. All women received a card that included information about referrals for violence. The midwives were blinded to which video the women watched, and although they received information about the content of the videos, the midwives did not watch the videos. The questionnaire and videos were linguistically translated by professional translators into Norwegian, English, Urdu, and Somali and culturally adapted during the development process by women with Norwegian, Pakistani, and Somali backgrounds (as described in the user-involvement study, which has been submitted for publication).

The aim of this qualitative study was to explore midwives’ attitudes towards a tablet intervention to prevent IPV and their experiences with recruiting participants with differing ethnic backgrounds to a study that used the tablet intervention.

## Methods

### Interviews

Semistructured individual interviews with 9 midwives who recruited participants for the Safe Pregnancy study were conducted by LG (qualitative researcher) or TK (master’s student in midwifery). LG and TK had not met the participating midwives prior to the interviews. Interviews were conducted between June 2019 and September 2019, approximately at the same time when recruitment for the Safe Pregnancy study was finalized at the MCHCs. Interviews were conducted in Norwegian. Of the 9 interviews, 7 interviews took place at the midwives’ worksites and 2 at the researchers’ worksites. The interviews lasted from 14.00 minutes to 53.38 minutes. The interviews followed a semistructured interview guide (Multimedia Appendix 1) that was developed by the interdisciplinary research group of the Safe Pregnancy study. The interview guide was pilot tested with a midwife who recruited participants for the Safe Pregnancy study. TH conducted the pilot interview in the presence of LG. The pilot test did not lead to any substantial amendments in the final interview guide. The pilot interview was included in the final analysis. The main themes in the interview guide were midwives’ motivation to participate in the Safe Pregnancy study, midwives’ attitudes toward a tablet intervention to promote safety behaviors, and midwives’ experiences with including pregnant women of different ethnic backgrounds.

### Recruitment

Midwives were purposely recruited by LG and TK from the 19 participating MCHCs in the Safe Pregnancy study [27]. The recruitment strategy was to recruit midwives from MCHCs that varied in the cultural backgrounds of their users and number of recruited women. Recruitment was carried out until a rich set of individual cases was reached.

In total, 9 midwives were asked to participate, and none of the midwives refused to participate. Midwives who helped to recruit women in the Safe Pregnancy study participated in individual teaching sessions on the use of the tablet, how to assess eligibility, and how to recruit women. In addition, midwives had several opportunities for professional development related to IPV, such as attending an international conference about IPV during pregnancy and project workshops with a mix of presentations from various resources from the field of violence against women. The Regional Committee for Medical and Health Research Ethics approved the study (ref.nr: 2017/358), and participants gave their written consent to participate.

### Analysis

Interviews were audiotaped and transcribed by LGH and TK. LGH, LH, TK, and ML read the transcripts. LGH randomly compared some of the transcripts with the audiotapes to ensure the accuracy of the transcription process. The analysis was guided by thematic analysis, according to Braun and Clarke [28], and included the following steps: (1) familiarizing themselves with the data by repeated reading of each informant’s transcripts, (2) generating initial codes (words or short phrases in the transcripts) that were relevant for the research questions, (3) organizing the codes into subthemes, (4) arranging the subthemes into overarching themes, and (5) defining and naming the themes. LGH and TK conducted the analysis and discussed potential codes and themes with the other authors. A qualitative software program, HyperRESEARCH 4.0.2 (ResearchWare Inc, Randolph, MA), was used to identify codes and systematize subthemes. In the presentation of the results, midwives are given fictitious names to secure their anonymity.

## Results

### Characteristics of the Midwives

All the midwives worked at the MCHCs, and they routinely asked women about their experiences with violence. Midwives were 42-57 years old. Their length of experience working in prenatal care at MCHCs was 4-18 years. One midwife recruited at 2 MCHCs.

The analysis resulted in 3 themes representing the midwives’ attitudes toward a tablet intervention and their experience with recruiting for the Safe Pregnancy study (Textbox 1). The first theme, motivation to participate in the Safe Pregnancy study, represents both the midwives’ motivation to participate as well as their general experiences when they asked women about IPV in prenatal care. The second theme, attitudes toward a tablet intervention, describes the midwives’ perceived advantages and disadvantages of the use of a tablet during prenatal care to present information about IPV and a safety-promoting video. The third theme, experiences with the recruitment, presents the midwives’ general experiences with including women, their suggestions to improve the recruitment process, and their experiences with including women of different ethnic backgrounds.

**Themes and subthemes for the midwives’ attitudes toward a tablet intervention and experience with recruiting for the Safe Pregnancy study.**

**Theme 1: Motivation to Participate in the Safe Pregnancy Study**

- Need for more training and advice for communication about intimate partner violence (IPV) in pregnancy
- Need for more knowledge about IPV in general
- Implementation of a routine enquiry for IPV in pregnancy
- Being part of a network
- Contributing to research

**Theme 2: Attitudes toward a tablet intervention**

- Intervention could act as an anonymous “door-opener” to talk about IPV
- Intervention could make women aware that they experience IPV
- Trust in the content of the tablet intervention
- Importance of face-to-face communication

**Theme 3: Experiences with Recruitment**

- Engaged to recruit
- Time-consuming and disturbing
- Facilitators to recruit
- Experiences including women with different ethnic backgrounds
- Linguistic barriers and skepticism toward scientific research

### Midwives’ Motivation to Participate in the Safe Pregnancy Study

For many of the midwives, their motivation to participate in the Safe Pregnancy study was their perceived need for more advice and training about how to ask pregnant women about IPV in order to help them. For example, Anna, who had worked at an MCHC for about 4 years, described her motivation to participate as the following: “It’s to get more knowledge and to be more confident to ask.”

This subtheme emerged especially when midwives perceived it challenging to communicate about IPV. These midwives felt that they needed better skills to communicate about IPV to help women and hoped that they could improve their skills through the information in the Safe Pregnancy study’s workshops.

Others considered themselves experienced with talking about IPV with pregnant women and did not perceive it as challenging (Textbox 2).

**Example of an exchange between a midwife and moderator.**

You have to find the key for each woman. Every woman has her own key.

Sara (Midwife)

And do you feel that you succeed in finding these keys?

Moderator

Yes.

Sara

Midwives who did not perceive it challenging to communicate about IPV were motivated to participate in the study because they were interested in the general knowledge about IPV provided in the workshops, such as the different kinds of violence, adverse health outcomes, and how they could help women.

Midwives often stated that their motivation to participate was the implementation of the national guidelines that strongly recommend midwives ask their clients about IPV, as described by Sandra who had worked long-term in prenatal care: “All of these guidelines came about that we had to ask everybody about violence…and I felt a little bit like ‘how do we do that?’”

They were also motivated to become part of a network where they could discuss their experiences and get support from peers. Another motivator to participate was that they wanted to contribute to new scientific knowledge about how to ask about IPV and how to promote safety behaviors during pregnancy.

### Midwives’ Attitudes Toward a Tablet Intervention to Inform About Intimate Partner Violence and Promote Safety Behaviors

The majority of the midwives had positive attitudes toward the use of a tablet during prenatal care to provide information about IPV as well as a video to promote safety behaviors, as described by Sandra who worked at an MCHC where many participants were recruited:

No, I think that, to answer anonymously on a tablet, that’s a nice way to answer nowadays. Because I think that it is difficult to answer this question face-to-face.

Sandra

They perceived prenatal care as a good opportunity to use the tablet intervention to promote safety behavior, because as Alexandra stated, “it may help that women are in a safe environment.”

Midwives considered a video promoting safety behaviors on a tablet as an appropriate tool for both ethnic Norwegian women and women of different ethnic backgrounds, given that the video is available in various languages. The opportunity for women to answer questions about IPV anonymously and look at safety behaviors on their own was the most frequently mentioned advantage of the tablet intervention. In addition, midwives perceived that the tablet intervention could serve as a door-opener to talk with the women about IPV, as explained by Monica, who had been working for a long time in an urban MCHC with a multiethnic user population:

If the video says something like ‘your midwife will talk about this and that it is important that she knows, because she has a duty to confidentiality and she can find out how to help you,’ (…) That can get people to open up easier. Maybe not the first time one asks, but maybe at a later consultation.

Monica

Even though the midwives had not seen the video, they thought that visual information about the different forms of IPV could more easily make women aware that they are victims of violence, compared with face-to-face consultations. Several midwives found it challenging that they had not seen the video and stated that it would be important that they could trust the video content and that the information in the video is in line with the information they provide to women. In this context, midwives said that a potential advantage of the video on a tablet was the possibility to ensure that every woman gets the same information, as stated by Alexandra: “Then you are sure that everyone gets the same [information], maybe.”

Many midwives thought that the video could serve as an appropriate supplement to their care and outlined that it would be important that women were followed up face-to-face:

I really believe in the face-to-face conversation, with a safe midwife, talking individually…), but one can make people aware about things, in such a video, but it demands a follow-up.

Ida

Even though midwives mentioned several advantages of the tablet intervention, some were skeptical since IPV was perceived as a sensitive issue. Alexandra, who had worked at an MCHC for several years, said:

But I think that there is no easy solution for this. It’s not like that we just can show them a video about it and everything is solved. I don’t think so.

Alexandra

They said that it would be important to build trust prior to asking women about their experiences with IPV.

### Midwives’ Experiences With Recruiting Women for the Safe Pregnancy Study

In general, midwives appeared to have been very engaged in the recruitment of pregnant women and could even imagine asking women to participate in the Safe Pregnancy study as a door-opener to ask them about IPV. Midwives thought the name of the project, “Safe Pregnancy,” might have helped to include women, as it was a general term and not too specific about violence, as described by Sara who also had recruitment experiences from other research projects: “No, I didn’t think that it was especially challenging to recruit for a research project about violence, because we should not say that the project was specifically about violence, but that it is about safe pregnancy.”

Even though midwives were engaged to recruit, the following statement illustrates that they sometimes felt that they had not succeeded including women who they thought had experienced IPV:

Yes, some women are like, you don’t really come close to them. I don’t know how to explain. But there are women where you think that they should have been included. It’s not sure that they have experienced some kind of violence, that’s what I don’t know, but, yes…

Alexandra

Some midwives perceived the recruitment process as disturbing and time-consuming. Midwives explained that they had to deal with many health-related issues in a very short consultation time and having to include women in the Safe pregnancy study disturbed the work. The required time for the intervention was often related to technical problems, usually problems with the internet connection and gaining access to the intervention. Assistance from health secretaries to keep watch for the tablets as well as the availability of separate rooms where participants could be alone with the tablet helped to overcome time-related problems with the recruitment. Some midwives also wanted to have more support and regular follow-up visits by the researchers.

Some midwives experienced challenges including women of different ethnic backgrounds. As expressed by Monica, who recruited at an MCHC with a multicultural population, some midwives found that it was easier to include ethnic Norwegian women: “There have been more nonethnic Norwegian women saying no to participating…”

Midwives reported that well-integrated immigrant women were more willing to participate and mentioned limited language and reading skills as the main barriers to their inclusion. One midwife said that women from Pakistan and Somalia preferred the Norwegian version of the intervention, as they could not read their mother tongue. Midwives thought that the availability of more languages would have made it easier to recruit more women of different ethnic backgrounds.

Another barrier to including women of different ethnic backgrounds was that the midwives often experienced that women of other ethnic backgrounds were skeptical toward research, as described by Anna who has worked at an MCHC with a multicultural user population: “Regarding the questionnaires, for example, women had commented that ‘that’s not how we talk about these issues in our culture.’”

## Discussion

### Principal Findings

Midwives in this study perceived a tablet intervention as an appropriate supplement to provide information about IPV and to promote safety behaviors during prenatal care. Given the sensitivity of IPV, midwives outlined the importance of following up the intervention with face-to-face communication. Midwives reported technical problems and high time demands as the main challenges to recruiting women. They experienced some challenges including immigrant women due to linguistic barriers and the women’s skepticism about scientific research.

### Comparison With Previous Work

The midwives’ main motivation to participate in the Safe Pregnancy study was to get more information about IPV and to improve their skills for communicating about IPV to help women. In other studies, health professionals also reported barriers to face-to-face communication about IPV in clinical settings [20,21,29-31]. Discomfort with questioning women about IPV, a fear of offending women, and uncertainty about management after disclosure are the main barriers in face-to-face communication [18,32]. Ongoing mHealth interventions to assess IPV and promote safety behaviors show promising results for overcoming some of the barriers regarding face-to-face communication [22,23,26]. The tablet intervention in the Safe Pregnancy study was perceived as an anonymous door-opener to talk about IPV and provided a good opportunity to increase the women’s awareness of violence. A computer tablet intervention was also perceived as a safe and confidential way for abused women to disclose IPV without fear of being judged in a US-based RCT during perinatal home visits [22]. In addition, Glass et al [26] developed and tested a computerized aid to improve the safety decision process in an ethnically diverse sample of abused women in the United States. The aid improved the decision process as demonstrated by reduced decisional conflict after only one use.

As already mentioned, little is known about health professionals’ and women’s attitudes toward mHealth interventions to promote safety behaviors in order to reduce IPV. Even though the midwives in our study had positive attitudes toward a tablet intervention, they perceived it as an important supplement to face-to-face communication. In line with previous studies, the midwives said that they first had to build trust with the women prior to asking them about IPV [22,31,32]. Women who experienced violence reported a good relationship with the midwife and the trustworthiness of the midwife as a facilitator to talk about IPV [19]. In addition to a trustworthy patient-provider relationship, Bacchus et al [22], who tested an mHealth intervention during perinatal home visits with women experiencing IPV, outlined the importance of considering the clinical context in which interventions for IPV are embedded. The midwives in our study sometimes considered the intervention to be time-consuming and disturbing. Thus, more research is needed about the feasibility of mHealth interventions in prenatal care to promote safety behaviors.

Interventions addressing IPV must consider the cultural and social context where the intervention is implemented [33]. However, communication about IPV seems to be especially challenging between health professionals and women of culturally diverse backgrounds [19]. mHealth offers the opportunity to provide tailored information for different ethnic groups [34]. The Safe Pregnancy study was available in Norwegian, Somali, and Urdu and was culturally adapted during the development process by women with Norwegian, Pakistani, and Somali backgrounds. However, the midwives in our study experienced challenges including immigrant women due to limited language skills and their skepticism toward research projects. This agrees with other studies reporting challenges including women of different ethnic backgrounds [35,36]. Lopez-Class et al [35] identified strategies to recruit immigrant participants. Customizing incentives to specific ethnicities and involvement of local community organizations relevant to immigrants were identified as the most relevant. More research is needed to overcome the barriers related to including immigrant women who have experienced IPV.

### Strengths and Limitations

To our knowledge, this is the first study to investigate health professionals’ attitudes toward a tablet intervention during prenatal care to educate women about IPV and promote safety behaviors. The participating midwives represented 10 of the 19 recruitment sites of a large RCT among a multicultural study population; one of the midwives worked at 2 MCHCs. The study included a small sample size, which is typical for qualitative studies [28]. This method was chosen since qualitative studies can contribute to the complex evaluation of mHealth interventions [17]. Only one interview was conducted by LG and TK together, which might have influenced the interviews because of their different educational background and interview style. However, the transcripts and potential themes in the analysis were discussed among all the authors to improve the credibility of the findings [37].

### Conclusions

This study shows that the midwives perceived a video tablet intervention during prenatal care as an appropriate supplement to educate women about IPV and promote safety behaviors. Although the video was considered as an anonymous door-opener to talk about IPV, midwives perceived it important to follow the intervention with face-to-face communication with the women. The scarcity of midwives’ time during consultations and technical problems have to be considered when implementing the intervention during prenatal care. Further research is needed to overcome the barriers to including women of different ethnic backgrounds.

## Appendix

Multimedia Appendix 1 Supplementary file (interview guide).

## Abbreviations

IPV: intimate partner violence.
MCHC: mother and child health center.
mHealth: mobile health.
RCT: randomized controlled trial.
