# Supplementary material for: Midwives’ Attitudes Toward and Experience With a Tablet Intervention to Promote Safety Behaviors for Pregnant Women Reporting Intimate Partner Violence: Qualitative Study
Source: JMIR Mhealth Uhealth. 2020 May 20;8(5):e16828. doi: 10.2196/16828 (PMC7270855; doi:10.2196/16828)
Supplement: Multimedia Appendix 1 [file mhealth_v8i5e16828_app1.docx]

**Interview guide: Midwives’ Attitudes toward and Experience of a Tablet Intervention to Promote Safety Behaviors for Pregnant Women of Different Ethnic Backgrounds Reporting Intimate Partner Violence**

Information about the aim of the study and research ethics

Introduction question: “How long have you been working in antenatal care?”

Themes with specific questions:

1. Motivation for participating in the Safe Pregnancy study

- What was your main motivation to participate in the Safe Pregnancy study?
- How have you been working with IPV prior to the study?
- Can you give me some examples?
- How would you describe your knowledge of the national guidelines for prenatal care that outline to ask women routinely about IPV?

1. Attitudes toward a tablet intervention to promote safety behaviors

- Did you have any strategies to talk about IPV prior to this study?
- Can you give me some examples?
- What do you think about a video to promote safety behaviors on a tablet in prenatal care?
- Can you think about alternative supporting aids to talk about IPV?

1. Experiences to recruit pregnant women of different ethnic backgrounds

- Can you describe your experiences to recruit women for the Safe Pregnancy study?
- How did you experience to recruit women of different ethnic backgrounds?
- What do you think motivated women to participate?
- What do you think facilitated successful recruitment?
- Have you experienced any barriers in the recruitment?
